# A New Whooping Cough Vaccine That May Prevent Colonization and Transmission

**DOI:** 10.3390/vaccines5040043

**Published:** 2017-11-10

**Authors:** Michael J. Brennan

**Affiliations:** 1 Aeras, 1405 Research Blvd., Rockville, MD 20850, USA; MJ.Brennan45@gmail.com; Tel.: +1-301-648-0155; 2 Iliad Biotechnologies, 230 East 15th Street, Suite 1-A, New York, NY 10003, USA

**Keywords:** *Bordetella pertussis*, whooping cough vaccine, BPZE1

## Abstract

This article is a Letter to the Editor. The major purpose of this Letter is to highlight the development of a new genetically altered whooping cough vaccine. Recently a baboon model has been used to show that this next generation pertussis vaccine can prevent colonization, as well as disease, and elicit antibodies against major pertussis antigens. Two phase I clinical trials have been performed, showing that this new vaccine is safe in humans, and a phase II trial will be performed in the US in 2018.

During this season, the shadows of violence and war have engulfed the world, and hurricanes have devastated the Caribbean, impacting inhabitants who have little access to medical care or immunizations. There is a need for scientists and others to focus on positive and promising events. One is the development of a next generation vaccine for whooping cough. At first, this seems like a step backwards, but the whole-cell pertussis vaccine has always been effective, and now, with the genetic removal of three toxins in BPZE1, it should be safer.

In the 1980s dissatisfied parents and others were unhappy with the adverse events that often accompanied the whole-cell whooping cough (pertussis) vaccine routinely given to their children. The U.S., Europe and other countries were motivated to develop a safer pertussis vaccine. A worldwide [1] alliance of researchers, manufacturers and governments was formed, along with political backing, to find new pertussis vaccines. In the U.S., this included a union of the NIH, FDA and CDC with pharmaceutical companies and clinicians to test and choose such vaccines. In a relatively short time, new acellular pertussis vaccines were tested in large phase III clinical trials in several countries, including Japan, Sweden, Italy and Senegal, and novel acellular pertussis vaccines were licensed in a number of developed countries by the early 1990s. It took more time for the complete schedule of vaccinations to be licensed, and only some, mostly industrialized, countries adopted the new vaccines.

Unfortunately, severe outbreaks of whooping cough have occurred since; for example, in 2012 there were 48,277 cases in the U.S., the highest in 50 years. The reasons for this are complex (see ref. [2] for examples), but it includes evidence that acellular vaccines are less effective than expected over the time necessary for protecting humans against *Bordetella pertussis*. Also, they do not prevent infection and transmission of the causative pertussis agent [3]. A new vaccine for whooping cough is much needed as a primary and/or boost.

A new live attenuated pertussis vaccine, BPZE1, in which three toxins—including the pertussis toxin—have been genetically modified or removed has been developed in the laboratory of Camille Locht at the Institut Pasteur de Lille. Mouse studies have provided evidence of protection against challenge [4] and, more recently, work performed in a baboon model of pertussis [5] has demonstrated that the BPZE1 vaccine is exceptional in that, upon nasal administration, it transiently colonizes the nasopharynx. BPZE1 not only prevents pertussis disease in animals but also *Bordetella pertussis* colonization, a feature that resembles the protection observed after recovering from the disease. This is very important, as current vaccines do not prevent colonization by the bacteria [3]. In two Phase I human clinical studies, BPZE1 has shown excellent safety, and elicits antibody levels against the major pertussis antigens [6]. Efficacy as a prime or boost vaccine awaits further clinical study and evidence that this new vaccine can be manufactured in quantity, reproducibly. A phase II clinical trial of BPZE1 will be performed in the U.S. in 2018, and will provide more information, including additional efficacy data [7]. Together, current studies indicate that BPZE1 can prevent colonization, pertussis disease and ultimately may block transmission, which could halt whooping cough in both young children and adults. If successful in the clinic, global introduction of BPZE1 could, in time, eliminate whooping cough.
